# Receptors and Cofactors That Contribute to SARS-CoV-2 Entry: Can Skin Be an Alternative Route of Entry?

**DOI:** 10.3390/ijms24076253

**Published:** 2023-03-26

**Authors:** Manon Barthe, Leslie Hertereau, Noura Lamghari, Hanan Osman-Ponchet, Véronique M. Braud

**Affiliations:** 1 Institut de Pharmacologie Moléculaire et Cellulaire, Université Côte d’Azur, CNRS UMR7275, 06560 Valbonne, France; manon.barthe@pkderm.com (M.B.); hertereau@ipmc.cnrs.fr (L.H.); lamghari@ipmc.cnrs.fr (N.L.); 2 PKDERM Laboratories, 45 Boulevard Marcel Pagnol, 06130 Grasse, France

**Keywords:** SARS-CoV-2, skin, ACE2, TMPRSS2, cathepsin L, NRP1, furin, CD147, AXL, KREMEN1, ASGR1

## Abstract

To prevent the spread of SARS-CoV-2, all routes of entry of the virus into the host must be mapped. The skin is in contact with the external environment and thus may be an alternative route of entry to transmission via the upper respiratory tract. SARS-CoV-2 cell entry is primarily dependent on ACE2 and the proteases TMPRSS2 or cathepsin L but other cofactors and attachment receptors have been identified that may play a more important role in specific tissues such as the skin. The continued emergence of new variants may also alter the tropism of the virus. In this review, we summarize current knowledge on these receptors and cofactors, their expression profile, factors modulating their expression and their role in facilitating SARS-CoV-2 infection. We discuss their expression in the skin and their possible involvement in percutaneous infection since the presence of the virus has been detected in the skin.

## 1. Introduction

Coronaviruses are a diverse family of enveloped positive-sense single-stranded RNA viruses that infect not only humans but also other mammalian and avian species, including livestock and pets. They are therefore considered a challenge for public health as well as a veterinary and economic concern [1,2].

Generally speaking, human coronaviruses cause seasonal and commonly mild respiratory tract infections associated with “cold” symptoms. In contrast, severe acute respiratory syndrome coronavirus (SARS-CoV) and Middle East respiratory syndrome coronavirus (MERS-CoV) which have emerged in the human population over the past 20 years, are highly pathogenic [3].

**Figure 1:**
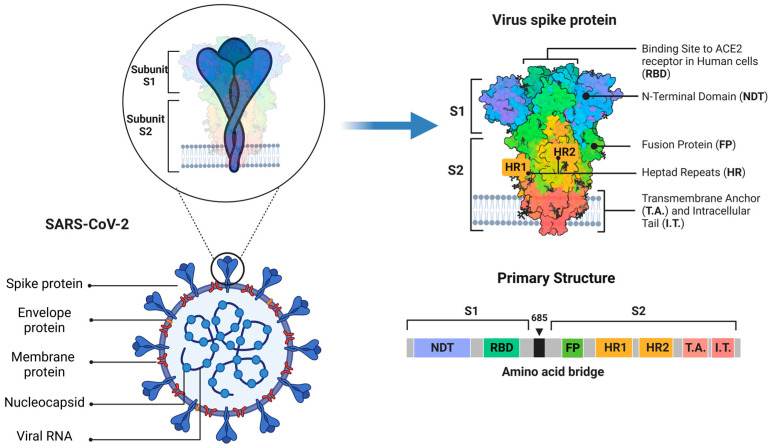
Overall structure of SARS-CoV-2 virus and tridimensional and primary structures of the spike protein.

SARS-CoV-2 is a novel severe acute respiratory syndrome coronavirus that emerged in Wuhan, Hubei, China in December 2019, and then rapidly evolved into a global pandemic affecting millions of people within a short period of time [4]. This respiratory disease manifests with fever, pneumonia and can progress to severe, life-threatening respiratory pathology and lung damage for which limited specific prophylactic or therapeutic treatment has been approved to date [3,4]. The enveloped virus contains a positive-sense single-stranded RNA genome with a size of ~26–32 kilobase (kb) and a nucleocapsid (N) with helical symmetry of ~120 nm. The viral envelope consists of a lipid bilayer, where the viral membrane (M), envelope (E) and spike (S) structural proteins are anchored [5,6] (Figure 1).

SARS-CoV-2 has been shown to be transmitted by close contact via exposure to infected droplets and aerosols [7]. The rapid rate of spread of SARS-CoV-2 and transmission via the respiratory tract (with the identification of the nasal epithelium as the initial gateway of infection) has led the World Health Organization to recommend drastic protection measures. Such measures include repetitive/frequent hand-washing, as well as the application of hydroalcoholic gel, gloves and face masks. As a result, the implementation of these drastic measures to control infection have greatly fragilized human skin. Self-administered online questionnaires of frontline healthcare workers in Hubei, China, revealed an overall prevalence rate of 97.0% of skin lesions caused by the enhanced infection prevention measures [8,9]. The most affected sites are the nasal bridge, hands, cheeks, and forehead and the symptoms mainly include skin dryness/tightness, itching, tenderness, burning/pain, in addition to skin lesions such as desquamation, erythema, papules, epidermal maceration, fissures, erosion, among others [8,9]. As skin is a functional physical and immune barrier that prevents the invasion of foreign pathogens, including bacteria, fungi, and viruses [10], such measures triggering skin disruption may result in percutaneous transmission. Not to mention that the virus has been found to survive for 72 h on plastic or stainless steel, 24 h on cardboard and 9 h on human skin [11,12]. Importantly, recent studies have revealed differences in viral stability and disinfection efficacy between the Wuhan strain and all variants of concerns [13]. Briefly, on plastic and on skin surfaces, Alpha, Beta, Delta and Omicron variants exhibited more than twice the survival time of the Wuhan strain, with the Omicron variants having the longest survival time. Specifically, the survival times of the Wuhan strain, Alpha, Beta, Gamma, Delta and Omicron (BA.1 and BA.2) variants on skin surfaces were 8.6 h, 19.6 h, 19.1 h, 11.0 h, 16.8 h, 21.1 h and 22.5 h respectively. In addition, in vitro analyses of disinfectant efficacy showed that the Alpha, Beta, Delta and Omicron variants were slightly more resistant to ethanol than the Wuhan strain [13]. Such differences underscore the importance of gaining a thorough understanding of the mechanisms of transmission and virus entry into cells in order to control the pandemic. Global tissue tropism for viruses is determined by the availability of virus receptors and entry cofactors in host cells. Therefore, in this review, we describe in detail the SARS-CoV-2 receptors and cofactors reported to date, their overall tissue expression and the evidence supporting their importance in SARS-CoV-2 infection, including the controversies regarding some of them. Finally, we also address the issue of skin, which is in contact with the viruses, and is affected by the stringent sanitary measures taken since the beginning of the pandemic. We discuss the evidence that question the skin as an alternative route of entry for SARS-CoV-2, with the risk of percutaneous transmission of the virus via compromised skin.

## 2. Mechanisms and Pathways of SARS-CoV-2 Entry into Host Cells

It is the spike (S) protein of coronaviruses that facilitates viral entry into target cells. The SARS-CoV-2 spike protein is composed of two subunits, named S1 and S2. The S1 subunit, known as the receptor binding subunit, contains the N-terminal domain (NTD) and the Receptor Binding Domain (RBD). The S2 subunit, on the other hand, is known as the membrane fusion subunit and is composed of the Fusion Peptide (FP) and two heptad repeats (HR1 and HR2) that are structural units that function in membrane fusion (Figure 1) [14].

The initial steps of infection involve specific binding of the RBD located in S1 to the peptidase domain of the cell entry receptor **angiotensin-converting enzyme 2 (ACE2) [5,15].** Some evidence in the literature has indicated that NTD is also involved in the entry of most coronaviruses, including SARS-CoV-2, but its functional role has yet to be fully defined [16,17]. The S protein undergoes cleavage at two different sites. First, at the boundary/interface between the two S1 and S2 subunits (S1/S2 site), the proprotein convertase **furin** separates the RBD and fusion domains. The second cleavage occurs at the S2′ site after binding to ACE2 and involves cellular proteases: **transmembrane serine protease 2 (TMPRSS2)** or endolysosomal **cathepsin L (CTSL)**, further exposing the fusion peptide to facilitate the fusion of the virus and host cell membrane [18,19,20]. Recently, ADAM17 and ADAM10 were found to also cleave the spike protein and facilitate SARS-CoV-2 infection [21]. **Neuropilin-1 (NRP1)** is known to bind furin-cleaved substrates and has been found to bind to furin-cleaved S1 protein, facilitating viral entry via endocytosis, thereby potentiating SARS-CoV-2 infectivity [22,23].

Additional receptors that represent alternatives to ACE2 for SARS-CoV-2 cell entry have been identified. They include **CD147**, **AXL**, **KREMEN1**, **ASGR1, CD209 (CLEC4M), CLEC4G, transferrin receptor or TIM1.** They may play a significant role, particularly in cells or organs where ACE2 is not expressed. Their role as attachment receptors or as cofactors facilitating viral entry has been shown but for most it remains to fully assess whether they are biologically relevant [6].

Once binding of SARS-CoV-2 has occurred, fusion of the virus with the host plasma membrane usually occurs within acidified endosomes, which is followed by the formation of a funnel like structure between HR1 and HR2, facilitating fusion and subsequent release of the viral genome into the cytoplasm [6]. This is followed by replication of the viral genome, and synthesis of the subgenomic RNA of the viral structural proteins (M, E and S proteins). These genes are then translated, inserted into the endoplasmic reticulum (ER) and move along the secretory pathway into the ER-Golgi intermediate. Finally, the interaction between the viral M and E proteins is required to form Virus-Like Particles (VLPs), suggesting that these two proteins function together to produce coronavirus envelopes [5,6] (Figure 1).

## 3. SARS-CoV-2 Primary Receptor and Cofactors

**Figure 2:**
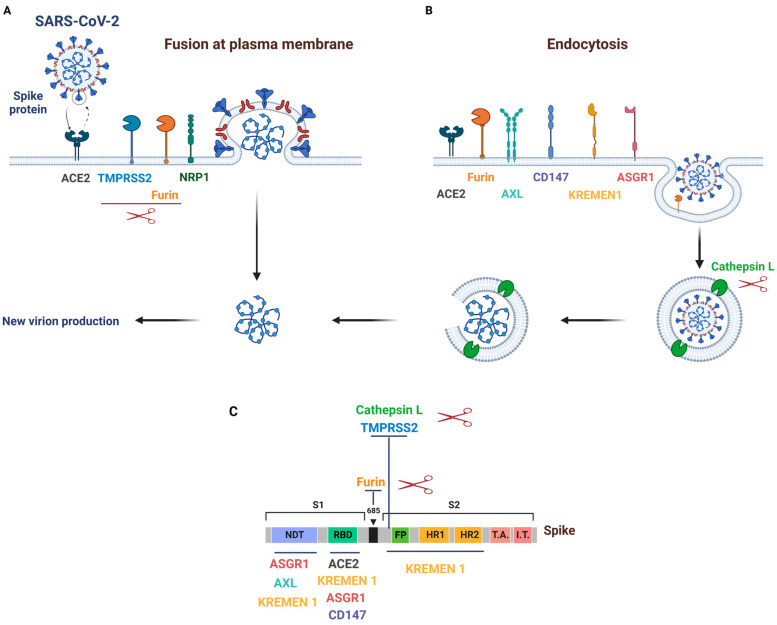
Main receptors and cofactors of SARS-CoV-2, adapted from [24]. Viral entry relies on two mechanisms: (**A**) fusion of the virus with the plasma membrane, mechanism ACE2 and TMPRSS2 dependent, (**B**) entry of the virus by endocytosis, mechanism TMPRSS2 independent and involving cathepsin L. Entry by endocytosis depends on binding to ACE2 or ACE2-independent receptors AXL, CD147, KREMEN1 and/or ASGR1. (**C**) Mapping of the binding sites of SARS-CoV-2 receptors on the spike protein.

Like SARS-CoV, SARS-CoV-2 uses ACE2 as its primary host entry receptor but it also uses host factors to potentiate its infectivity [6]. Below, we review what is known about ACE2 and its cofactors (Figure 2).

### 3.1. ACE2

#### 3.1.1. Background

Angiotensin-converting enzyme 2 or ACE2 was initially identified in 2000 as a homolog of the ACE receptor [25,26]. Its gene is located on the X chromosome (chromosomal location Xp22) and is composed of 18 exons and 19 introns generating 6 variants by alternative splicing. ACE2 is a type I transmembrane protein of 805 amino acids with a single N-terminal extracellular domain containing the catalytically active site domain with a conserved HEXXH zinc-binding metalloprotease motif, and a C-terminal membrane anchor domain. ACE2 acts as a carboxypeptidase by removing a single amino acid from the C-terminus of its substrates [25,26].

Under normal conditions, ACE2 is localized on the plasma membrane with the N-terminus containing the catalytic site protruding into the extracellular space, thus using as substrates different active peptides present in the interstitium [27]. ACE2 is involved in blood pressure regulation, being a key regulator of the renin–angiotensin–aldosterone system. Unlike ACE which cleaves angiotensin I to angiotensin II, inducing vasoconstriction, ACE2 degrades angiotensin II to angiotensin I-7, which binds Mas/G coupled receptors that protect against cell death. Angiotensin II degradation also induces vasodilation [28]. The protective role exerted by ACE2 may be lost upon binding of SARS-CoV-2, which triggers ACE2 shedding.

#### 3.1.2. ACE2 Tissue Expression and Its Importance in SARS-CoV-2 Infection

Studies on tissue expression of ACE2 began around the year 2000 when this protein was initially discovered as an ACE homolog. Initial Northern blot experiments indicated that ACE2 mRNA can be detected in human heart, kidney and testis, suggesting a possible role in cardio-renal function [26]. Harmer et al. [28] then confirmed these data and conducted additional qRT-PCR experiments to further clarify tissue expression of ACE2. Their results revealed that ACE2 mRNA is detected in many tissues. They showed that ACE2 is expressed in tissues of the gastrointestinal tract, including the ileum, duodenum, jejunum, caecum and colon, with the ileum showing the highest expression [28].

At the protein level, Donoghue et al. [25] showed by Immunohistochemistry (IHC) that ACE2 protein is found mainly in the heart (specifically in the endothelium of most intramyocardial vessels including capillaries, venules and medium-sized coronary arteries and arterioles), as well as the kidney (in the entire endothelium, focally in the rare smooth muscle cells of medium-sized vessels and also in the epithelial cells of the proximal tubule).

The study of ACE2 tissue expression received increased attention when it was found to be the functional receptor for the novel coronavirus, SARS-CoV [15]. Hamming et al. [29] analyzed ACE2 protein expression by IHC in 15 human organs including oral and nasal mucosa, nasopharynx, lung, stomach, small intestine, colon, skin, lymph nodes, thymus, bone marrow, spleen, liver, kidney and brain. In brief, they found that ACE2 is present in arterial and venous endothelial cells and arterial smooth muscle cells in all of the organs studied, which is consistent with previous reports [28]. This localization may explain the micro-vasculopathy observed in COVID-19 patients, associated with a loss of pericytes consecutive to apoptosis [30]. The most interesting finding was the surface expression of ACE2 protein on lung alveolar epithelial cells and small intestine enterocytes, cells that are in contact with the external environment. These data provided a better understanding of the possible pathways of entry of SARS-CoV and were a first step in understanding the pathogenesis of the main manifestations of the disease, especially in the lungs. Interestingly, in the skin, Hamming et al. detected expression of ACE2 in the basal layer of the epidermis and in smooth muscle cells surrounding sebaceous glands, with strong expression in eccrine gland cells [29].

During the 2019 SARS-CoV-2 outbreak, high-throughput sequencing techniques such as single-cell RNA sequencing (scRNA-seq) enabled a comprehensive and accurate analysis of the expression level and distribution of ACE2 mRNA in human tissues at a single cell level. While ACE2 expression levels in the whole lung are intermediate, high expression was reported in type II pneumocytes [31,32]. Zou et al. [33] explored the published scRNA-seq datasets from various tissues and organs of different human body systems, including the respiratory system (nasal mucosa, respiratory track, bronchus and lung), the cardiovascular system (heart), the digestive system (esophagus, stomach, ileum and liver) and the urinary system (kidney and bladder). They used the expression level of ACE2 in alveolar type II cells (AT2) cells as a reference (as it is generally accepted that 2019-nCoV tends to attack lung AT2 cells via the host receptor ACE2) [32]. Based on eight individuals, they calculated that ACE2-positive AT2 cells represented 1% of total lung cells. They therefore stratified the organs studied into high-risk (>1% ACE2^+^) and low-risk (<1% ACE2^+^) organs [33]. In brief, the high-risk organs were the lungs (due to AT2 cells) as well as the airways (ACE2 being primarily expressed by epithelial cells), esophagus, heart, bladder, kidney and ileum. They also examined available data from protein databases to strengthen their findings. The data obtained showed that ACE2 protein is enriched in the small intestine enterocytes and renal tubules, as well as in lung alveolar epithelial cells, cardiac cells, arterial smooth muscle cells, and the gastrointestinal system [33]. Another study by Li et al. [34] analyzed RNA-Seq datasets of normal human tissues from the UCSC Xena project and found that the lung and adrenal gland expressed medium levels of ACE2 and the skin expressed lower levels. The highest levels were detected in small intestine, testis, kidney, heart, thyroid and adipose tissue.

Interestingly, in agreement with the gastrointestinal tract expressing the highest levels of ACE2, enterocytes appeared to be an active replication site for SARS-CoV-2 [35]. However, the study by Triana et al. [36], while confirming SARS-CoV-2 infectivity in human intestinal epithelial cells from human intestinal organoids, did not find a correlation between susceptibility to infection and ACE2 expression level. They observed that SARS-CoV-2 infection is associated with downregulation of ACE2 expression in the human gut [36].

Modulation of ACE2 expression has been further investigated. Ziegler et al. [31] showed that the release of inflammatory cytokines, such as interferons (IFNs), specifically type I IFNs, and to a lesser extent type II IFNs, upregulates ACE2 in human airway epithelial cells [31]. This stimulation was confirmed by Zhuang et al. [37] who detected an increase in mRNA upon infection with several viruses such as influenza virus, rhinovirus or other coronaviruses, and upon IFN-β stimulation in human bronchial epithelial cells. Of note, they also found moderate and high increase of ACE2 in human primary keratinocytes stimulated with IFN-α and IFN-γ respectively, suggesting that the virus may have the potential to bind to skin cells. Chua et al. [38] also described an upregulation of ACE2 in infected epithelial cells. In human colon organoids, IFN-γ induced the differentiation of enterocytes, increased ACE2 expression and their susceptibility to SARS-CoV-2 infection [39]. The conclusion that ACE2 is an IFN-stimulated gene is now toned down by the identification of a variant named dACE2 which encodes a truncated protein that no longer binds to the RBD domain of SARS-CoV-2 S protein. dACE2, but not ACE2, was upregulated upon IFN stimulation in primary human bronchial epithelial cells and human intestinal organoid cultures [40]. Consistent with this finding, two other reports identified a short ACE2 induced by IFN that lacks the ability to bind to S protein [41,42]. Aside from inflammatory conditions, ACE2 and also TMPRSS2 expressions are regulated by the androgen receptor and this may explain why men seem to be more sensitive to the virus [43].

Finally, it has been shown that ACE2 can be shed by several proteases: disintegrin and metalloproteinase domain-containing protein (ADAM)10, ADAM17, and transmembrane protease serine 2 (TMPRSS2) [21]. Cleavage of ACE2 by ADAM17/tumor necrosis factor-converting enzyme (TACE) occurs at ectodomain sites and a soluble form, which retains its catalytic activity (termed sACE2) is produced. In contrast, TMPRSS2 promotes proteolytic cleavage of ACE2 at the intracellular C-terminal domain, and does not produce sACE2 [27].

In the case of SARS-CoV infection, it has been shown that the virus invades human cells via this ACE2 receptor [15] and that TMPRSS2 and ADAM17 compete for ACE2 cleavage but only processing by the former promotes SARS-CoV Spike protein (SARS-S)-driven entry [44]. This highlights the importance of not only ACE2 as the primary pathway for SARS-CoV-2 entry, but also of other cofactors that play a central role in facilitating SARS-CoV infection. SARS-CoV-2 can enter cells via two pathways, i.e., by host membrane bound peptidases or by endocytosis. Below, we review the proteases that have been identified as playing a role (Figure 2).

### 3.2. TMPRSS2

#### 3.2.1. Background

TMPRSS2 is a transmembrane serine protease 2 encoded by the *TMPRSS2* gene on chromosome 21q22.3, which is widely conserved and has two isoforms, both of which are autocatalytically activated from the inactive zymogen precursor protein [45]. The TMPRSS2 protein is a 70 kDa type II transmembrane serine protease structurally composed of an N-terminal cytoplasmic domain, a transmembrane domain, and a C-terminal extracellular domain comprising a class A LDL receptor domain, a cysteine-rich scavenger receptor domain and an activation domain linked to a serine protease domain by a disulfide bond [46]. Like other serine proteases, TMPRSS2 has been associated with physiological and pathological processes such as digestion, tissue remodeling, blood coagulation, fertility, inflammatory responses, tumor cell invasion, apoptosis and pain [45]. TMPRSS2 cleaves at single arginine or lysine residues. During infections with influenza and coronaviruses, it activates viral fusion proteins at so called monobasic cleavage sites [20].

#### 3.2.2. TMPRSS2 Tissue Expression and Its Importance in SARS-CoV-2 Infection

With respect to tissue expression, TMPRSS2 is widely expressed in epithelial cells of the respiratory, gastrointestinal, and urogenital tracts [20,45,47]. In addition, TMPRSS2 has been shown to be highly expressed in bronchial epithelial cells compared to surfactant-producing type II alveolar cells and alveolar macrophages, and not expressed in type 1 alveolar cells that form the respiratory surface [47]. TMPRSS2 expression is also present in the liver and in the skin [47]. It has been shown to be highly expressed in human prostate epithelium and regulated by androgen hormones, AR-responsive elements being present in its promoter [45]. Indeed, TMPRSS2 was initially identified in prostate cancer and TMPRSS2 was shown to be strongly upregulated in response to androgens in prostate cancer cell lines [47]. Similarly, androgen administration to a lung adenocarcinoma cell line upregulated TMPRSS2 transcripts [48] and AR signaling inhibition reduced ACE2 and TMPRSS2 expression levels and SARS-CoV-2 viral entry [43]. These data strongly suggest a crucial role of ARs in regulating TMPRSS2 expression.

Importantly, coronaviruses as well as influenza viruses are critically dependent on TMPRSS2 for viral entry and propagation in the host [19]. This correlates with its expression in lung epithelial cells [24,49,50]. As previously mentioned, and speaking exclusively of coronaviruses, following binding of S protein to the ACE2 receptor, cleavage by furin, the S protein is cleaved by TMPRSS2. Studies by several teams have revealed that TMPRSS2-deficient mice do not develop severe symptoms when infected with influenza A virus strains, SARS-CoV and MERS-CoV, due to inhibition of viral proteolytic activation and subsequent viral spread along the respiratory tract, confirming the crucial role played by TMPRSS2, in mediating respiratory viral infections [19,20,51,52,53,54]. On intestinal organoids, Triana et al., [36] also showed that SARS-CoV-2 genome copy numbers in human intestinal epithelial cells correlated with the expression level of TMPRSS2. They hypothesized that TMPRSS2 may play a more important role than ACE2 in the cell tropism of SARS-CoV-2. Interestingly, a higher incidence of severe COVID-19 was reported in males. One hypothesis is that it is related to the strong regulation of TMPRSS2 by androgens [43,47]. TMPRSS2 was also found to be upregulated on primary airway epithelial cells and Calu-3 upon TLR5 stimulation by *P. aeruginosa* flagella. TMPRSS2 induction by flagellin depends on p38 and NF-kB and increases infectivity of SARS-CoV-2 [55].

Taken together these observations led to postulate that direct or indirect targeting of TMPRSS2 could be a relevant therapeutic approach to prevent, limit and treat SARS-CoV-2 infection. Antagonists of androgen receptor signaling could be repurposed to benefit COVID-19 patients [56]. Inhibitors specifically selected to inhibit TMPRSS2 activity are being tested [57]. Recently, such an inhibitor has been developed and characterized. It is a peptidomimetic compound named N-0385 [58]. This compound specifically inhibits TMPRSS2 activity and inhibits SARS-CoV-2 infection both in vitro in human lung epithelial cells and colonoids and in vivo using an established mouse model of severe SARS-CoV-2 disease and intranasal administration.

### 3.3. Furin

#### 3.3.1. Background

Furin is a subtilisin-like peptidase encoded by the *FURIN* gene on chromosome 15. Also known as the paired basic amino acid residue-cleaving enzyme (PACE), furin is a 794-amino-acid ubiquitous calcium-dependent proprotein convertase composed of a N-terminal signal peptide, an inhibitory prodomain, a catalytic endopeptidase domain, a P domain and a cysteine-rich domain linked to a transmembrane domain and a C-terminal cytoplasmic domain [59]. Furin has been shown to cleave precursors of a wide range of proteins at a preferred consensus sequence Arg-X-Arg/Lys-Arg↓-X and is thus involved in many normal and pathological processes [20,59]. Importantly, furin has been identified as an activating protease for the fusion proteins of a broad range of viruses, including highly pathogenic avian influenza A viruses (HPAIV), HIV, Ebola virus, measles virus, yellow fever virus and SARS-CoV-2, as well as bacterial toxins such as Shiga toxin or anthrax toxin at multibasic motifs [60].

#### 3.3.2. Furin Tissue Expression and Its Importance in SARS-CoV-2 Infection

Furin mRNA is ubiquitously expressed and detected at higher levels in liver, lung, brain, thyroid gland, prostate pancreas, skin, spleen and salivary glands [50,59]. Furin has been associated with COVID-19. The distinguishing feature of SARS-CoV-2 from SARS-CoV and SARS-related CoVs is the presence of a proprotein convertase cleavage site at the boundary between the S1/S2 subunits, which is cleaved during biogenesis specifically by furin [14,18]. Sequence analysis of the SARS-CoV-2 S protein indeed confirmed that the S1/S2 site of SARS-CoV-2 S protein contains an insertion of four amino acids (R-R-A-R685↓) providing a minimal proprotein convertase cleavage site in contrast to the S protein of SARS-CoV [20]. The role of furin was highlighted by Hoffman et al. [61] who showed that SARS-CoV-2 S protein processing was blocked in a concentration-dependent manner in the presence of decanoyl-RVKR-CMK, a furin inhibitor. In addition, blocking SARS-CoV-2 S protein cleavage at the S1/S2 site abolished viral entry into TMPRSS2(+) cathepsin B/L-dependent S protein activation pathway (low) human lung cell line (calu-3), but had no effect on entry into TMPRSS2(−) cathepsin B/L-dependent kidney cell line (Vero E6 cells). Their data collectively confirmed that a multibasic S1/S2 site is essential for SARS-2-S-driven entry into human lung cells, a characteristic feature of furin cleavage [61]. Similar results were observed in human airway organoids, where a furin cleavage site in SARS-CoV-2 S protein was found to increase infectivity. In addition, the work of Shang et al. [14] also highlighted the importance of furin cleavage during SARS-CoV-2 cell entry. Briefly, using pseudotyped virus entry assays on three different types of target cells (human cervical, lungs and lung fibroblast cells), they proved, using siRNA, that furin is responsible for this cleavage, while ruling out the possibility of indirect furin-dependent activation of matrix metalloproteinases (MMPs), which in turn could activate the SARS-CoV-2 S protein. Collectively, their data showed that furin is the proprotein convertase that preactivates the SARS-CoV-2 spike during viral packaging [14]. More recently, Peacock et al. [62] generated a recombinant SARS-CoV-2 with mutations at the furin cleavage site and showed that this disrupted infection of TMPRSS2-expressing cell lines and transmission in a ferret infection model. They also demonstrated that furin cleavage allows escape from the anti-viral activity of IFITMs in the endosomal compartment [62]. Taken together, these data emphasize the importance of TMPRSS2 and furin in SARS-CoV-2 S protein activation and suggest that these mechanisms may allow the virus to avoid the endosomal/lysosomal pathway, an alternative pathway of entry that exhibits innate mechanisms limiting virus replication. This issue is addressed below in the description of cathepsin L.

### 3.4. Cathepsin L

#### 3.4.1. Background

Cathepsin L (CatL) is a lysosomal cysteine endopeptidase encoded by *CTSL* gene on chromosome 9. It is composed of a heavy chain linked by a disulfide bond to a C-terminal light chain. It is synthesized as an inactive preproenzyme in the ER where the signal peptide is cleaved, generating the procathepsin form, which travels through the Golgi to the endo/lysosomes while acquiring glycosylation [63]. Inside the lysosomes, procathepsin L is cleaved into a mature form by self-activation [64]. CatL is catalytically active at low pH and preferentially cleaves peptide bonds with aromatic residues at P2 and hydrophobic residues at P3 [63]. It is involved in many biological and pathological processes.

#### 3.4.2. Cathepsin L Tissue Expression and Its Importance in SARS-CoV-2 Infection

Cathepsin L (CatL) is a cysteine protease that is expressed in all tissues and cell types. Proteolytic functions occur primarily in the endo/lysosomal compartments, but CatL is also released in the cytosol, nucleus, mitochondria or extracellular space to perform specific functions. In endo/lysosomes, CatL contributes to antigen presentation by degrading the invariant chain involved in the folding of MHC II molecules [65]. It is also involved in innate immunity by cleaving TLR7 and TLR9 ectodomains, in autophagy, and in various cellular processes such as neural development, fertility [64]. Impaired CatL activity induces the accumulation of α-synuclein amyeloid fibrils. Upon oxidative stress or lysosomotropic agents, permeabilization of the lysosomal membrane releases CatL in the cytosol or nucleus where it participates to processes such as apoptosis, inflammation and cell cycle regulation [66]. In addition, CatL has also been shown to be involved in tumor invasion and metastasis, to participate in extracellular matrix remodeling, in atherosclerosis, kidney disease, diabetes, bone disease, or viral infection [64]. In the context of SARS-CoV-2 infection, a role for CatL has been demonstrated alongside TMPRSS2 and furin [14,19]. Originally, CatL was found to be an important activating protease for SARS-Co-V infection, cleaving at the S1–S2 boundary [67,68]. The cleavage site is conserved in SARS-CoV-2 and treatment with ammonium chloride which blocks CatL inhibited pseudotyped virus replication into cell lines [19]. Ou et al. also demonstrated a role of CatL in virus entry [69]. A genome-wide CRISPR knockout screen identified *CTSL* gene as one of the required host factor for SARS-CoV-2 infection [70].

The importance of CatL in SARS-CoV-2 infection was further highlighted by the work of Zhao et al. [71]. Briefly, they analyzed, for the first time, circulating CatL levels in COVID-19 patients and found that these levels were indeed elevated after SARS-CoV-2 infection and also positively correlated with disease progression and severity [71]. For this reason, and since there is no treatment available for SARS-CoV-2, there is a greater focus on studying compounds known to inhibit CatL activity. One example is the work performed by Smieszek et al. [72], who performed high-throughput drug screens and discovered that amantadine hydrochloride, originally used to treat influenza A, not only reduces CatL expression but also disrupts the lysosomal pathway, interfering with the virus ability to replicate. Other examples of such compounds have been reviewed elsewhere (see [66]). Among them, chloroquine has been tested as it increases the pH of endo/lysosomes and thus inhibits cathepsins. However, it does not inhibit infection of TMPRSS2-expressing cells because virus entry is independent of CatL [73]. It is crucial to note, however, that cathepsin inhibitors exert toxicity in cells, and that broad-spectrum inhibition of CatL may result in unpredictable side effects due to its pleiotropic functions and involvement in normal physiological processes [66].

### 3.5. NRP1

#### 3.5.1. Background

Neuropillins (NRPs) are transmembrane, glycoproteins present in all vertebrates and highly conserved between species. They include NRP1 and NRP2 which share 44% homology and genes are located on human chromosomes 10 and 2, respectively [74]. They are composed of a large N-terminal extracellular domain divided into three domains with distinct functions: one binding to class III semaphorins, one binding to VEGF and one involved in oligomerization. This extracellular domain is linked to a transmembrane domain and a short cytoplasmic PDZ domain [74,75]. Multiple splice variants have been identified, some of which encode soluble forms that exhibit decoy functions. Both proteins were initially discovered as neuronal adhesion molecules involved in Semaphorin-mediated axonal guidance [75]. However subsequent studies have identified a wide range of ligands for NRP1 and NRP2 and revealed that they are in fact multifunctional proteins that regulate pleiotropic biological processes, including cardiovascular and neuronal development, such as axonal guidance, angiogenesis, vascular permeability, and bone homeostasis. In addition, they have been shown to play a major role in immunity and tumorigenesis as reviewed in [76,77].

#### 3.5.2. NRP1 Tissue Expression and Its Importance in SARS-CoV-2 Infection

NRP1, like NRP2, is ubiquitously expressed, particularly in the central nervous and vascular systems. NRP1 is also expressed on subsets of immune cells [76]. In homeostasis, NRP1 also called CD304/BDCA4 is a marker of plasmacytoid dendritic cells (DC). NRP1 has also been found expressed on multiple immune cell subsets in various physiological and pathological situations, such as expression on mature myeloid DC, macrophages, effector and memory T cells, Treg, Tfh and NKT cells [76]. Importantly, NRP1 is abundantly expressed in the respiratory and olfactory epithelium, with higher expression in endothelial and epithelial cells. It is expressed in the suprabasal layers of the epidermis in human skin and is correlated with the degree of differentiation [78]. In the context of SARS-CoV-2 infection, it has been shown that the C terminus of the SARS-CoV-2 S1 protein generated by furin cleavage has an amino acid sequence with a [R/K]XX[R/K] motif consistent with the “C-terminus rule” (CendR) [79]. This motif is recognized by NRP1 and NRP2 and mediates endocytosis [80]. Several viruses use NRPs as viral entry factors. Work by Daly et al. [23] and Cantuti-Castelvetri et al. [22] confirmed the interaction of the furin-cleaved SARS-CoV-2 S1 protein with NRP1. Both teams also showed that NRP1 potentiates and enhances SARS-CoV-2 entry and infection, in the presence of ACE2 and TMPRSS2, consistent with the role of NRP1 as co-receptor [22,23]. Finally, analysis of scRNA-seq data showed that NRP1 RNA expression levels were elevated in SARS-CoV-2 positive bronchial epithelial cells compared with SARS-CoV-2 negative cells and adjacent bronchoalveolar lavage fluid (BALF) cells from severely affected COVID-19 patients [22]. In human lung tissue and human olfactory epithelium, ACE2 was detected at very low levels while NRP1 was highly expressed, and SARS-CoV-2 S protein could be detected in NRP1^+^ cells. Thus, NRP1 might represent one of the cofactors required to facilitate virus–host cell interactions, especially in cells with low ACE2 expression. Interestingly, S protein may compete with physiological ligands for binding to NRP1 and cause some of the dysfunctions reported in COVID-19 patients such as loss of olfaction, analgesic effects, immune interference and modulations of signaling pathways [80].

## 4. SARS-CoV-2 Alternative Receptors

With the exception of ACE2, little is known about other host receptors that SARS-CoV-2 might use to enter cells. However, the low levels of ACE2 in the airway, which are restricted to a minor cell population and the multi-organism tropism of SARS-CoV-2 strongly suggest that it is rather unlikely that ACE2 is the only cellular host receptor [6]. Here, we detail other receptors that have been described to bind to SARS-CoV-2 and could play a significant role in COVID-19 (Figure 2).

### 4.1. CD147

#### 4.1.1. Background

Cluster of differentiation 147 (CD147), also referred to as basigin or extracellular matrix metalloproteinase inducer (EMMPRIN), is a transmembrane glycoprotein of the immunoglobulin superfamily encoded by *BSG* gene located on chromosome 19. CD147 is composed of an extracellular N-terminal domain containing two Ig-like domains, a transmembrane domain and a cytoplasmic C-terminal domain. Homodimers can be found in cis and trans. Glycosylation of the extracellular domain is required for its function [81]. It has been identified as the main upstream regulator of matrix metalloproteinases (MMPs) [82].

#### 4.1.2. CD147 Tissue Expression and Its Importance in SARS-CoV-2 Infection

CD147 is widely expressed in human tissues and exhibits heterogeneous glycosylation between tissues and cell types. It is upregulated in chronically inflamed mucosa and in cancer [83]. Importantly, CD147 expression is regulated by various transcription factors, miRNA and soluble mediators in different physiological, pathological and tissue-specific contexts. Homophilic and heterophilic interactions with a variety of binding partners result in CD147 being involved in multiple biological functions reviewed elsewhere [84,85]. CD147 is also involved in pathological processes such as tumor development, plasmodium invasion, bacterial and viral infections [86,87,88]. CD147 was previously found to interact with cyclophilin A-associated SARS-CoV N protein, facilitating SARS-CoV infection [89]. The authors evaluated whether CD147 could also facilitate SARS-CoV-2 infection. They found that the S protein RBD bound to CD147 with a K_D_ = 1.85 ×10^−7^ M [87]. The interaction was confirmed by co-immunoprecipitation and also visualized by electron microscopy in SARS-CoV-2-infected Vero E6 cells and in lung and kidney tissues. Knockdown of CD147 reduced virus copy number, while its overexpression promoted infection. Two studies disputed these results [90,91], showing that full length or RBD spike did not bind to CD147 and that knocking down or blocking CD147 did not decrease viral infection. Interestingly, both used cell lines expressing high level of ACE2 while a third study confirmed a role of CD147 in A459 lung cell line which express low level of ACE2 [92]. This may explain the discrepancies observed. It was shown that CD147 expression allowed SARS-CoV-2 to enter into otherwise non-susceptible cell lines and the virus-induced cytopathic effect was inhibited by Meplazumab, a humanized anti-CD147 (IgG2) monoclonal antibody in a dose-dependent manner [87]. Interestingly, the authors excluded a role for ACE2, which does not bind to CD147. They also showed that immune T cells that do not express ACE2 were infected via CD147 endocytosis and inhibited by Meplazumab. Although peripheral blood mononuclear cell (PBMCs) are not the primary cells infected with SARS-CoV-2, these results suggest that their infection via CD147 may contribute to the pathology. An exploratory phase 2 study showed that the Meplazumab significantly reduced symptoms and duration of COVID-19 in patients suggesting that CD147 plays a role in virus entry and/or immune modulation [93]. More recent studies demonstrated that CD147 is a universal receptor for Alpha, Beta, Gamma, Delta and Omicron SARS-CoV-2 variants and that anti-CD147 mAb inhibits infection and cytokine storm in preclinical mouse models [94,95].

A complementary study on the role of CD147 in SARS-CoV-2 infection, performed by Fenizia et al. [88] showed that CD147 plays a different role in mediating SARS-CoV-2 infection compared to the original SARS-CoV. That is, in SARS-CoV infection, CD147 interacts with its ligand cyclophilin A (CyPA) to facilitate viral entry. In the case of SARS-CoV-2 infection, Fenizia et al. [88] showed that CD147 knockdown in cell lines reduced the abundance of ACE2 protein but not its RNA, meaning that CD147 directly or indirectly affects SARS-CoV-2 infection via its ability to regulate ACE2 abundance at the post-translational level. Finally, they compared the expression levels of both CD147 and ACE2 in SARS-CoV-2-infected and uninfected cell lines. Their data showed that the activities of these two receptors in SARS-CoV-2 entry are co-regulated, as their expression is downregulated upon exposure to the virus (at the RNA and protein level), indicating that viral infection acts at the transcriptional level [88]. Clearly, more needs to be learnt to fully depict the role of CD147 in facilitating SARS-CoV-2 infection.

### 4.2. AXL

#### 4.2.1. Background

Phosphatidylserine receptors have been shown to facilitate the binding and internalization of a wide range of viruses [96]. Two members of the T-cell immunoglobulin and mucin domain (TIM) family (TIM-1 and TIM-4) and one of the Tumor-associated macrophage (TAM) family (AXL) via Gas6, are particularly effective [97]. AXL is a receptor tyrosine kinase encoded by a gene on chromosome 19 in humans which has been shown to regulate a variety of functions, including survival, growth, aggregation, migration and anti-inflammation in multiple cells. AXL, by binding to its ligand GAS6 which then interacts with phosphatidylserine on apoptotic cells or viruses, regulates innate immune responses [97]. In addition, overexpression and increased activity of AXL have been attributed to a number of chronic pathological conditions, including cancer and cardiovascular disease [98,99].

#### 4.2.2. AXL Tissue Expression and Its Importance in SARS-CoV-2 Infection

AXL is widely expressed with an onset of expression in late embryogenesis [100]. It is particularly expressed in bone marrow stroma, myeloid cells and within the respiratory and gastrointestinal tracts and reproductive tissues. Regarding its importance in SARS-CoV-2 infection, a study by S. Wang et al. suggested that AXL is a novel candidate receptor for SARS-CoV-2 [101]. A second study by Bohan et al. [102,103] confirmed this finding but disputed the exact role of AXL. Wang et al. [101] speculated that there are additional receptors or co-receptors for SARS-CoV-2 in addition to ACE2, as it shows very low level of expression in the lungs. Using SARS-CoV-2 S protein as bait, they analyzed protein complexes in pulmonary and bronchial cells by tandem affinity purification (TAP)-mass spectrometry (MS) [101]. Interestingly, they were unable to identify ACE2 in these lung- and bronchial cell lines, indicating that ACE2 may not be the primary host receptor of SARS-CoV-2 in these cells due to its low expression levels. Nevertheless, they consistently identified ACE2 by this technique in ACE2-expressing cell lines. Overall, they were able to identify three candidate receptors from 524 membrane proteins identified, after computational screening which eliminated non-specific binders. The candidate receptors with the most favorable affinity scores were AXL, epidermal growth factor receptor (EGFR), and low-density lipoprotein receptor (LDLR) but only AXL co-localized with SARS-CoV-2 S protein. Thus focusing only on AXL, S. Wang et al. [101] showed that AXL interacted with the NTD rather than the RBD that interacts with ACE2. Further experiments, including the overexpression of AXL, revealed that it promoted SARS-CoV-2 entry as efficiently as ACE2 overexpression, while knocking-out AXL in lung cell lines significantly reduced SARS-CoV-2 infection. They found that AXL promoted SARS-CoV-2 infection in ACE2-KO cells, suggesting a role independent of ACE2 [101]. This conclusion differs from the study of Bohan et al. [102,103] finding that AXL promoted SARS-CoV-2 infection through interactions with virion-associated-phosphatidyl serine in an ACE2-dependent manner.

### 4.3. KREMEN1 and ASGR1

#### 4.3.1. Background

Kringle Containing Transmembrane Protein 1 (**KREMEN1**) (as well as KREMEN2) was originally discovered as a novel transmembrane receptor-like protein, containing an extracellular kringle domain, a poorly folded WSC domain and a pseudo-Ig-like CUB domain [104,105]. The gene located on chromosome 22 encodes a high-affinity receptor for the secreted protein Dickkopf1 (Dkk1) and lipoprotein receptor-related protein 6 (LRP6), which antagonizes/inhibits canonical Wnt signaling [106]. KREMEN1 has also been identified as a host entry receptor for a group of enteroviruses [107]. Asialoglycoprotein receptor-1 (**ASGR1**) also known as CLEC4H1, is encoded on chromosome 17 and is a prototypic C-type lectin (CLEC) receptor binding carbohydrates, a type II protein with cytoplasmic, transmembrane domains and an extracellular carbohydrate recognition domain. ASGR is composed of two subunits: ASGR1 is the major subunit and ASGR2 is the minor subunit. The primary function of ASGR1 is to mediate endocytosis and degradation of desialylated proteins and cells in the circulation. ASGR1 has been implicated in the clearance of activated lymphocytes, apoptotic cells and is the gate of entry for hepatotropic viruses [108].

#### 4.3.2. KREMEN1 and ASGR1 Tissue Expression and Its Importance in SARS-CoV-2 Infection

**KREMEN1** is ubiquitously expressed while **ASGR1** is expressed primarily by liver parenchymal cells. ASGR1 is also expressed on myeloid cells and was detected in the circulation and in human skin dermis [109,110,111]. Recently, Gu et al. performed receptor profiling by measuring the binding of the SARS-CoV-2 S protein extracellular domain (S-ECD) to a variety of membrane proteins expressed in HEK293T cells [112]. Their study showed that 12 membrane proteins specifically interacted with the SARS-CoV-2 S-ECD with diverse affinities and profiles, including the main receptor ACE2. The identified membrane proteins were ASGR1, CD207, CLEC4M, ERGIC3, FUT8, KREMEN1, KREMEN2, LILRB2, LMAAN2, MGAT2 and SIGLEC9 [112]. Interestingly, CLEC4M (also known as L-SIGN or CD209L) has been identified as also binding to SARS-CoV [113]. Of these 12 proteins, only ACE2, KREMEN1 and ASGR1 were able to mediate SARS-CoV-2 infection. While KREMEN1 bound all three ECDs of the S protein (i.e., NTD, the RBD and the S2 subunit), ASGR1 bound only to the NTD and the RBD [112]. KREMEN1 and ASGR1 were able to mediate SARS-CoV-2 entry independently of ACE2 both in vitro and in vivo. Entry could be blocked by antibodies directed against KREMEN1 or ASGR1. The investigators showed that the virus not only uses distinct combinations of ACE2/ASGR1/KREMEN1 receptors to enter lung and liver cell lines, but they also established a strong correlation between the expression of these three receptors and susceptibility to the virus [112].

### 4.4. Additional Receptors Facilitating Virus Attachment

Several C-type lectins including CD209/DC-SIGN, CD209L/L-SIGN/CLEC4M, CLEC4G, that are known to bind to numerous viruses, have been described as candidate receptors that facilitate SARS-CoV-2 attachment and entry [112,114,115,116]. They function as pathogen recognition receptors. They can interact with the spike protein and facilitate SARS-CoV-2 entry. One study demonstrated that it involves a mechanism of trans-infection [115]. As the expression of these lectins is measured in many cell types, including epithelial and endothelial cells or immune cells, they may actively participate in the spreading of SARS-CoV-2 in tissues. Another alternative receptor identified through a genome-wide CRISPR activation screen is LDLRAD3, a member of the scavenger receptor superfamily, recently identified as the receptor of Venezuelan equine encephalitis [116,117]. It is expressed by neurons, binds the spike NTD and enhances or decreases SARS-CoV-2 infection when respectively overexpressed or knockdown [116]. DPP4 is also expressed on astrocytes and can mediate SARS-CoV-2 infection [118]. These receptors may therefore be particularly important in the spreading of the virus in the brain. More recently, a large screening of protein arrays identified the interaction of SARS-CoV-2 spike with the estrogen receptor alpha, suggesting that the virus can modulate its signaling [119].

### 4.5. LRRC15, a Novel Receptor Binding SARS-CoV-2 Spike and Suppressing Viral Entry

#### 4.5.1. Background

Leucin rich repeat containing 15 (LRRC15) is a single transmembrane protein encoded by *LRRC15* gene located on chromosome 3. It is expressed at the plasma membrane and in vesicles. It is composed of 15 extracellular LRRs and has a very short cytoplasmic domain. Like other members of the LRR family, LRRC15 is involved in cell–cell and cell–extracellular matrix interactions [120,121]. LLRC15 has been described expressed in cancer, on cancer-associated fibroblasts. Single-cell RNAseq analyses of cells of the tumor microenvironment identified a population of myofibroblasts expressing high level of LRRC15 whose genes were associated with extracellular matrix remodeling and immunosuppression. The specific depletion of this population augmented the response to anti-PD1 immunotherapy and enhanced antitumor activity of infiltrated CD8^+^ T cells. TGF-β2 signaling is required for the development of LRRC15^+^ myofibroblasts [122].

#### 4.5.2. LRRC15 Tissue Expression and Its Importance in SARS-CoV-2 Infection

LRRC15 is found mostly expressed in skin, lymphoid tissues, cervix, breast, placenta and expressed more specifically by fibroblasts. LRRC15 expression was associated with inflamed conditions such as cancer, autoimmunity and inflammatory diseases [123]. Through a whole-genome CRISPR activation screening approach, LRRC15 was found to bind to SARS-CoV-2 spike [124,125,126]. This interaction was confirmed in vitro. The interaction involved RBD domain [125,126]. Interestingly, LRRC15 did not facilitate entry of the virus but it was found to inhibit infection of permissive cells. The authors postulated that LRRC15 could participate in the innate immune protection of tissues, besides other related molecules such as TLR. The binding to spike could sequesters SARS-CoV-2 virions and thus limit infection [124,125,126].

## 5. Skin as an Alternative Entry Route for SARS-CoV-2 Virus

Transmission by contact through human skin is considered an important risk factor in the spread of viruses and it is therefore relevant to assess whether percutaneous infection could occur in the context of the COVID-19. Protective measures such as frequent hand washing, application of hydroalcoholic gel, or wearing masks have accelerated the rate of skin injury, which may promote viral transmission [9]. Patients suffering from skin diseases with defective skin barriers and under immunosuppressive treatments may also be more at risk.

**Figure 3:**
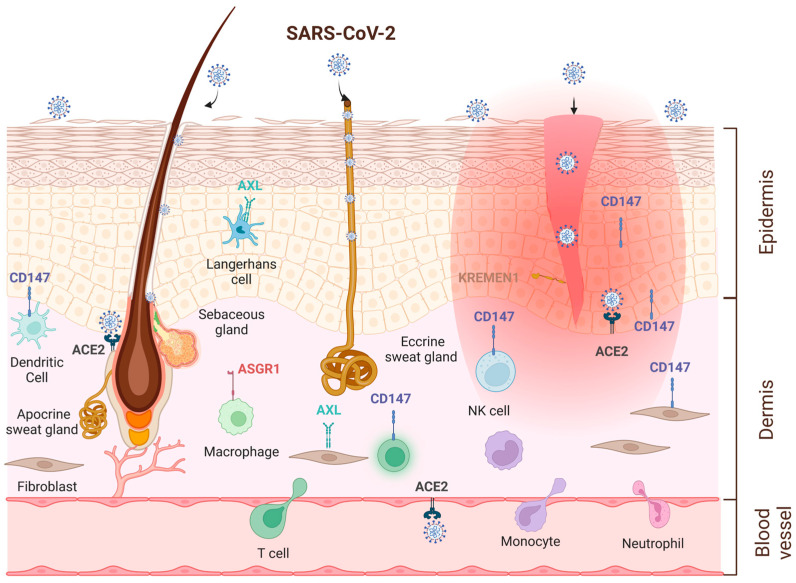
Possible routes of entry of SARS-CoV-2 virus in human skin: direct viral entry via skin lesions, sweat ducts or hair follicles or indirectly by deposition of circulating virus.

To infect skin cells, SARS-CoV-2 must meet several conditions. First, fomite transmission is required and has been measured, with approximately 16% or 9% of the virus transferred to human skin from wet or dry surfaces, respectively [127]. Second, the virus has to survive long enough on human skin, which is the case for all variants and omicron BA.2 is the most efficient, staying alive up to 22 h [13]. Third, SARS-CoV-2 cellular receptors and cofactors must be expressed by skin cells. Table 1 summarizes current knowledge on their expression profile in the skin. Fourth, the virus binds, penetrates and replicates into host cells. To do so, the virus needs to bypass the *stratum corneum* which functions as an efficient physical barrier, may enter via skin lesions, or hair follicles or sweat ducts and needs to develop viral mechanisms to interfere with cutaneous immune responses that are highly efficient at protecting against invading pathogens and also skin microbiome whose functions appear to be key in maintaining homeostasis and in inhibiting pathogenic growth [10,128] (Figure 3).

Many studies on COVID-19 report cutaneous manifestations associated with the disease. Initial dermatologic symptoms were reported in a single center observational study in Italy where approximately 20% of patients developed cutaneous manifestations such as erythematous rash, urticarial and chickenpox-like vesicles [129]. Another nationwide study in Spain found that chilblain-like lesions were associated with less severe COVID-19 cases whereas livedoid and/or necrotic lesions were associated to more severe ones [130]. Clinical reports have accumulated and it is now admitted that the five most common cutaneous abnormalities that are associated with COVID-19 are morbilliform rash, urticaria, vesicles, pseudo-chilblains and vaso-occlusive lesions [131]. Some of these symptoms such as skin rash are associated with positive COVID-19 swab testing and a UK study of 330,000 patients found that it was more predictive of infection than fever [132]. Interestingly, complement-mediated microvascular injury has been linked to severe COVID-19 in lung and cutaneous and subcutaneous microvasculature [133]. Micro-vasculopathy correlates with mild hyperplasia of pericytes in the skin [30] and is likely to be linked to the capacity of SARS-CoV-2 to infect endothelial cells [134]. Whether the dermal clinical manifestations are directly linked to SARS-CoV-2 infection of the skin or whether they are associated with larger deregulations triggered by the disease is however still unclear.

The presence of SARS-CoV-2 in the skin was demonstrated with the detection of viral spike protein in skin biopsies [131,133,135,136]. Cytoplasmic granular positivity for SARS-CoV-2 spike protein was detected in endothelial cells of the capillary venules in the dermis and in epithelial cells of the eccrine units of skin biopsies of patients with chilblains. Electron microscopy could also identify virus-like structures that resemble SARS-CoV-2 [134,135,137,138]. In one study, no viral RNA, only the spike protein could be detected in the microvasculature of the dermis and subcutaneous fat, questioning the presence of infectious viral particles and suggesting that pseudovirions could be released in the circulation and access the skin [135]. NP protein was detected in the epidermis of patients with COVID-19 but not in healthy skin [139]. Another case report was published where PCR was positive in skin but negative in the swab test, highlighting that skin infection may be more common than reported [140]. In addition, Liu et al. [141] analyzed skin autopsy samples from five COVID-19 patients and detected SARS-CoV-2 spike proteins in three of the five patients, primarily in sweat ducts and sweat glands and also in small blood vessels. Further analysis showed that the keratin Krt5^−^/Krt7^−^ epithelial cells and the Krt7^+^ secretory luminal cells were the major target cells in sweat ducts and glands respectively [141]. They also found co-expression of both ACE2 and TMPRSS2 with spike in sweat glands. All patients had vasculitis with lymphocyte infiltration, particularly adjacent to the epidermis and accessory glands in the dermis. These infiltrating cells were CD3^+^/CD8^+^ T cells and CD68^+^ macrophages but not CD4^+^ T cells, CD19^+^/CD20^+^ B cells or MPO^+^ neutrophils [141]. Using skin organoids, Ma et al. detected SARS-CoV-2 in KRT17^+^ hair follicles and neurons that are abundant in skin [136]. Chudakova et al. also reported an in vitro infection of 3D skin equivalents with SARS-CoV-2 [142]. By contrast, Zupin et al. could not infect 1D cultures of human keratinocytes and fibroblasts but they did not provide a positive control for the infection, questioning their results [143]. Alternatively, the lack of Infection of 1D cultures and the infection of 3D skin equivalents may suggest that the status of the skin cells is important to allow infection. Altogether, these observations underline the presence of the virus in skin and a possible role of the vascular system in the infection of the skin.

Consistent with possible dissemination into the skin, we can find evidence of expression of all the SARS-CoV-2 receptors and cofactors discussed above in the various cells comprising the skin. Indeed, the main receptor for entry ACE2 is transcribed at low levels in the skin (gene expression reported in genotype-tissue expression (GTEx) project (https://gtexportal.org) [144,145] but at higher levels in skin lesions of patients with psoriasis [146]. Single cell RNA sequencing revealed that ACE2 is mainly expressed in keratinocytes with increased expression in differentiating cells [139,147]. ACE2 is also transcribed in human subcutaneous adipose tissue, at a level higher than in total lung [148] and notably in endothelial cells of capillaries. Multiplex RNA in situ hybridization confirmed expression of ACE2 in keratinocytes and skin vasculature [149]. At the protein level, immunohistochemistry staining of healthy skin confirmed ACE2 expression in the basal and suprabasal epidermis, on keratinocytes, as well as in cells of the eccrine sweat glands [138,147,150]. Similar expression was found in skin from SARS-CoV-2 positive patients in which the spike protein was detected [138,139]. Interestingly, inflammatory conditions increase ACE2 expression [31,37,38,150,151]. ACE2 was found to be an IFN-inducible gene, although it is not clear whether the variant that is upregulated by IFN is functional, as recent studies demonstrated a specific upregulation of the short form of ACE2 which fails to bind to the spike protein [40,41,42]. ACE2 expression is also increased in skin from atopic dermatitis (AD) patients compared to healthy individuals and patients with psoriasis [150]. In vitro stimulation of keratinocytes with increasing concentrations of IL-33, a cytokine highly secreted by epidermal keratinocytes in AD led to the upregulation of ACE2 [150]. These results indicate that AD patients will be more susceptible to SARS-CoV-2 infection. This is coherent with the fact that not only ACE2 is expressed by skin cells, but also TMPRSS2, cathepsin L, furin, and NRP1 are found to be expressed (https://gtexportal.org) [47,78,139,144,145]. Cathepsin L is expressed at high level in inflammatory conditions such as AD, psoriasis, lupus erythematosus and cutaneous squamous cell carcinomas (cSCC) [152,153]. TMPRSS2 protein however may be expressed at a low level [138] or is more specifically found in sweat glands [141]. It remains to determine whether the virus favors the fusion membrane or endocytic pathway in skin tissue (Figure 2). TMPRSS2, like ACE2, is upregulated by AR which is expressed in the skin [47]. In skin diseases where AR is known to play a role [154], their upregulation might enhance virus transmission [43].

Besides ACE2, the other secondary receptors that may play a role in SARS-CoV-2 infection are also found in the skin. NRP1 is highly expressed on immune cells [77]. AXL is expressed on Langerhans cells and keratinocytes in human epidermis and upregulated in cSCC [155,156]. CD147 is expressed by keratinocytes, immune cells and upregulated in psoriatic skin lesions [145,157]. CD147 expression can be induced by cytokines such as IL-22, IL-1α, TGF-β1 or growth factors such as EGF [83,157,158]. As CD147 is a receptor mediating cellular entry of all the variants of SARS-CoV-2, it may play a preponderant role in the skin where it is highly expressed. ASGR1 is expressed in skin dermis by CD68^+^ macrophages and other unidentified cells. Knock-down of ASGR1 in macrophages enhanced the secretion of inflammatory cytokines in response to house dust mites [110]. ASGR1 is also expressed by monocytes in PBMCs and may therefore interact with virus in the circulation before being extravagated in the dermis [111]. KREMEN1 has also been detected in skin [159,160].

**Table 1:** Expression of SARS-CoV-2 receptors and cofactors in skin.

Receptors and Cofactors	Expression in Skin	References
ACE2	*mRNA*: low levels (whole skin)	https://gtexportal.org, [139,144,145,146,147,149]
	*Protein*: keratinocytes in the basal cell layer of epidermis, smooth muscle cells surrounding sebaceous glands, eccrine gland cells	[29,138,139,147]
	Upregulation by IFNs, IL-33, in AD, in psoriasis	[31,40,41,42,141,150,151]
TMPRSS2	*mRNA*: low levels (whole skin)	https://gtexportal.org, [47,139,144,145],
	*Protein*: sweat glands	[138,141]
Furin	*mRNA*: high levels (whole skin)	https://gtexportal.org,
	*Protein*: keratinocytes	[161]
Cathepsin L	*mRNA*: low levels (whole skin), higher in fibroblasts, immune cells	https://gtexportal.org, [159]
	*Protein*: low levels in healthy skin. Upregulation in AD, psoriasis, lupus erythematosus, cSCC	[152,153]
NRP1	*mRNA*: intermediate levels (whole skin), high in cultured fibroblasts	https://gtexportal.org,
	*Protein*: keratinocytes in the suprabasal cell layer of epidermis, immune cells	[77,78]
CD147	*mRNA*: high levels (whole skin), keratinocytes, fibroblasts, immune cells	https://gtexportal.org, [145]
	*Protein*: keratinocytes, fibroblasts, immune cells Upregulation by IL-22, IL-1α, TGFβ1, EGF	[157] [83,157,158]
AXL	*mRNA*: intermediate levels (whole skin), high in cultured fibroblasts	https://gtexportal.org,
	*Protein*: Langerhans cells, keratinocytes	[155]
KREMEN1	*mRNA*: intermediate/high levels (whole skin), expression on keratinocytes	https://gtexportal.org, [159,160]
	*Protein*: epidermis	https://www.proteinatlas.org
ASGR1	*mRNA*: low levels (whole skin) *Protein*: macrophages	https://gtexportal.org, [110,111]
LRRC15	*mRNA:* high expression in skin *Protein:* fibroblasts	https://gtexportal.org, [124,162]

## 6. Conclusions and Future Perspectives

As highlighted in this review, the entry of SARS-CoV-2 into human cells requires not only ACE2 but additional receptors and cofactors. However, the respective roles of each of them have not yet been fully characterized. This knowledge is needed to determine preventive measures and guide the development of therapeutic strategies and treatments.

The presence of receptors and cofactors for SARS-CoV-2 on healthy and compromised skin indicates that the skin may be an alternative route of entry that has been overlooked. On healthy skin, the virus can be efficiently eliminated by proper washing and use of hydro-alcoholic solutions, but in the context of the pandemic and the extensive use of protective measures, there is an increase in skin irritation and damage to the epidermal barrier, which allows the virus to access deeper layers of the skin. Patients already suffering from dermatologic diseases are particularly affected. This can result in inflammatory responses that upregulate SARS-CoV-2 receptors and cofactors. The outstanding question is whether the disruptions in skin homeostasis, skin microbiome and skin immunity affect virus entry and replication and, once the virus is present in the skin, the consequences, not only on skin immunity, but also on vascular, neural or hormonal systems remain to be fully assessed.

## Data Availability

Not applicable.
